# Theta band optogenetic stimulation of the septo‐hippocampal fibres in the fornix reduces interictal spikes and rescues memory performance in hAPP‐j20 mice

**DOI:** 10.1002/alz.088106

**Published:** 2025-01-03

**Authors:** Eva Vico Varela, James Eric Carmichael, Frédéric Manseau, Jisoo Choi, Guillaume Etter, Sylvain Williams

**Affiliations:** ^1^ Douglas Research Centre/ McGill University, Montreal, QC Canada; ^2^ MasGeneral Institute for Neurodegenerative Disease, Boston, MA USA

## Abstract

**Background:**

Altered neuronal timing and synchrony are biomarkers for Alzheimer’s disease (AD) and correlate with memory impairments. Electrical stimulation of the fornix, the main fibre bundle connecting the hippocampus to the septum, has emerged as a potential intervention to restore network synchrony and memory performance in human AD and mouse models. However, electrical stimulation is non‐specific and may partially explain why fornix stimulation in AD patients has yielded mixed results. We hypothesize that driving the specific circuits underlying rhythmicity within the fornix via optogenetics at the biologically relevant theta rhythm (5Hz) will stabilize septo‐hippocampal network timing in an AD mouse model (hAPP‐j20) to alleviate hypersynchronous interictal spikes (IS) and restore memory performance.

**Methods:**

GABAergic neurons in the septum were transfected with the excitatory opsin ChETA (or eYFP controls) in J20+/+ or J20± littermate controls. An optical fibre was placed above the fornix to limit excitation to the projection fibres and recording electrodes were placed in CA1 to measure network oscillations. 5Hz stimulation was applied continuously for 4 hours following the encoding phase of either novel‐object‐location or passive contextual avoidance memory tasks. We also combined *in vivo* calcium imaging with electrical recordings in CA1 to monitor network activity to determine how IS shape hippocampal cell assemblies in J20+/+ mice without fornix stimulation.

**Results:**

Interictal spikes were present in sleep in J20+/+ mice, and at a higher rate during REM (4.78/min) compared to slow‐wave (1.76/min; n = 11, p<0.001, one‐way ANOVA). J20+eYFP controls displayed a strong negative correlation between the rate of IS and memory recall score (n = 9, r = ‐0.62, p = 0.015). 5Hz optical fornix stimulation reduced the occurrence of IS in REM both during stimulation and 4hrs post‐stimulation (n = 11, p = 0.024, repeated measures ANOVA). Continuous 4‐hour theta stimulation between the encoding and recall phases of both memory tasks rescued J20+ChETA performance to levels comparable with healthy controls while J20+eYFP mice performed at the chance level (n = 9‐10 per group, p = 0.017, two‐way mixed ANOVA).

**Conclusion:**

These data validate theta band optical stimulation as a viable method to pace the septo‐hippocampal network effectively reducing IS events and recovering memory performance in an APP mouse model.

